# Behaviour of a Harbour Seal (*Phoca vitulina*) Mother and Pups in a Zoo Setting

**DOI:** 10.3390/ani16101545

**Published:** 2026-05-18

**Authors:** Susan C. Wilson, William Matthews

**Affiliations:** Tara Seal Research, Mablethorpe LN12 2AL, UK

**Keywords:** harbour seal, captive-born pup, mother–pup bond, body contact, body nosing, welfare

## Abstract

Although harbour seals have often bred successfully in captivity, there is little information on the behaviour and welfare of captive mothers and their pups. This study used video of a mother with her newborn pup (P1) during the first 16 days post-partum and further video of the same mother with a second pup (P2) during days 19–22. The most frequent behaviours in the water were body nosing and close body contact. The observations with pup P2 allowed for expanded descriptions of contact and following behaviours in the water. With P1, the mother displayed most responsibility for following and approaching, i.e., for maintaining contact. Suckling usually occurred immediately after the mother led the pup from the water to an onshore suckling location. P1 often rested alone while its mother checked on it continuously from the water and signalled it to return to the water by nudging, nosing and splashing at it. This mother engaged in close contact with both her pups, with both the mother and both pups displaying body language suggestive of positive emotion and, therefore, indicative of good welfare. These observations offer an argument in favour of allowing female harbour seals in captivity to give birth and wean their pups naturally.

## Simple Summary

A captive female harbour seal was video-recorded with her two pups born 13 years apart. The mother and pups displayed the same range of behaviours previously observed among mother–pup pairs around their nursery sites in the wild; in the water, these behaviours included the pups following their mother and continual nosing and body contact, all considered essential for primary pup socialisation. Recorded details of their underwater behaviour, seldom visible in the wild, helped to understand how mothers and pups maintain contact while travelling in the sea. During close contact in the water, the mother and both pups displayed body language suggestive of positive emotion and, therefore, indicative of good welfare.

## 1. Introduction

The harbour seal, *Phoca vitulina*, is unique among northern phocid seals in that full-term, precocial pups are born having already shed their lanugo, and are aquatically capable immediately after birth [1,2,3]. Nevertheless, births and nursery environments are shore-based, although mother–pup pairs may spend more than 60% of their time in the water [4]. From field observations, their aquatic activity has been described as consisting primarily of local movement within the intertidal area, involving the coordinated movement of mother and pup, including a strong following response by the pup, the young pup sometimes riding on its mother’s back, and constant chaperoning by the mother [1,5,6,7,8,9,10]. Frequent body contact between pairs has been noted when pairs linger together, sometimes playing, at the water’s edge [10]. Although suckling may occur in the water [1,11,12], it more typically occurs at the water’s edge preceding haul-out for a rest period [5,10,13]. Social interaction and contact between mother and pup while resting onshore are generally minimal [10].

In the wild, harbour seals inhabit two distinct environmental domains—(i) their haul-out sites and adjacent intertidal zone where they rest, give birth and interact socially; and (ii) the subtidal sea, through which they may travel many kilometres from their haul-out domain to offshore foraging sites [14]. When harbour seals are bred in captivity, their environment generally includes haul-out areas adjacent to a shallow pool of water, and a suitable enclosure design and dimensions can resemble some key aspects of the natural intertidal and onshore nursery habitats of wild harbour seal mother–pup pairs. Although the captive environment cannot mimic the offshore travelling and foraging environments of wild seals, it can mimic some physical aspects of their haul-out and nursery site habitats, although not the fish and crustacean fauna and benthic habitats of a typical intertidal zone—with an occasional notable exception [15].

Captive breeding of harbour seals has occurred frequently in zoos and aquaria throughout their range [16,17,18,19,20], while the closely related spotted seal, *Phoca largha*, has been captive-bred in China for conservation purposes [21]. Although these captive settings can potentially enable a range of natural mother–pup behaviours to be expressed, there has been very little published documentation on the behaviour and welfare of harbour seal mothers and their pups in zoos and aquaria [16,18,19]. There has been such documentation for other pinniped species in captivity—such as the phocid grey seal, *Halichoerus grypus* [22,23,24,25,26], the otariids, including California sea lion, *Zalophus californianus* [27] and Cape fur seal, *Arctocephalus pusillus pusillus* [28] and the Pacific walrus, *Odobenus rosmarus divergens* [29]; however, the natural patterns of maternal care, pup development and nursery habitats of these species differ in various respects from those of the harbour seal.

Where harbour seals have bred in captivity, there has sometimes been debate over the future of captive-born pups after weaning from their mothers. The options that have been practiced have included (1) retention of the pups in captivity [17], (2) euthanasia of the weaned pups on the grounds that they should not be released to the sea [20], and (3) permitted release of the pups into the sea [21]. Option 1 is only possible when there are vacancies for juveniles in the captive network, while option 2 raises grave welfare and ethical issues [20]. Option 3 raises potential problems for the welfare and species-normal behaviour of released pups; captive-bred spotted seal pups have been released at two years of age in a conservation effort to augment the wild population. However, post-release tracking has found that their at-sea movements are atypical of that population, likely compromising the conservation value of the release programme [21].

A zoo on the North Sea coast of eastern England has had a small breeding group of harbour seals for many years, with a policy of releasing any captive-born pups to sea within a few months of weaning. The present study uses archived and more recent video records of one mother (‘Victoria’) with two of her pups, born 13 years apart, to document key aspects of mother–pup behaviour in this zoo setting. Our hypothesis was that Victoria’s behaviour would encompass all the natural behaviours of wild mothers with their pups as they normally move around in and out of the water at their pupping sites. However, since observations of wild mothers caring for their newborn pups display behavioural flexibility according to the circumstances [3], behavioural adjustments appropriate to a captive environment were predicted. Furthermore, field observations have suggested some developmental changes in the mother–pup relationship as the lactation period progresses [5,7,8,30], and we therefore expected such changes to be evident in this captive setting. We also hoped that observation of the video footage might enable some new insights into harbour seal mother–pup behaviour from the opportunity to observe them underwater at a closer range than is possible in the wild. Finally, based on our observations of this captive mother with her pups and their comparison with wild mother–pup behaviour, we hoped to comment on captive mother–pup welfare and consider the outlook for post-weaning release of captive-born pups into the wild.

## 2. Animals and Methods

### 2.1. Animals and Captive Housing

**Figure 1:**
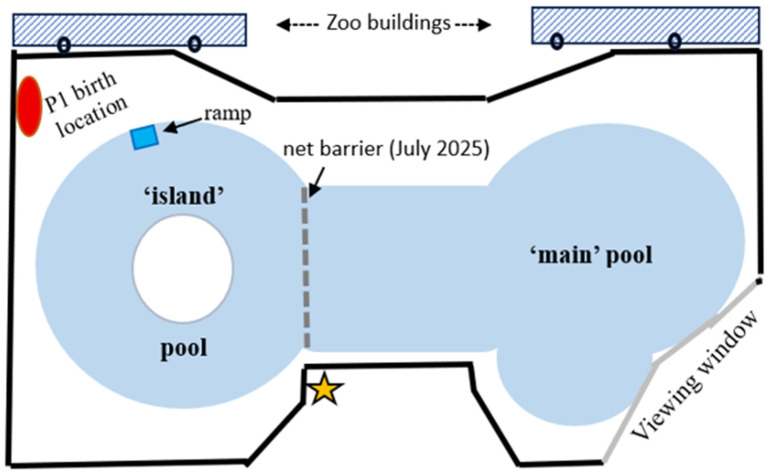
Schematic diagram of Natureland seal enclosure, including pool (21 m long, 9.4 m max. width, and 0.7–1.2 m depth range), surrounding haul-out platform, and temporary net barrier erected after pup P2’s birth in July 2025. Public viewing was restricted to outside the enclosure, bounded by a low wall (heavy black line). 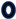 Approximate positions of CCTV cameras. 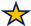 Observer location in July 2025.

The harbour seals inhabited an enclosure consisting of a pool 21 m long, containing 100,000 L filtered sea water pumped continuously throughout the pool, which was drained and cleaned weekly. The pool was surrounded by concrete platforms (referred to as ‘beaches’). The ‘island’ pool at one end, with an island at its centre, was ~8.9 m in diameter and ~0.7 m in depth. The ‘main’ pool at the opposite end was 9.4 m in diameter, ~1.2 m in depth, and bounded on one side by a glass viewing window (Figure 1).

The seal group consisted of animals originally admitted to ‘Natureland’ Zoo (Skegness, Lincolnshire, UK) as stranded orphan pups. Between 2006 and 2025, a total of 18 pups were born to this seal group, of which the eight who survived to weaning were hind-flipper tagged and released to the sea near large breeding colonies of harbour seals. This study focuses on the behaviour of one mother ‘Victoria’ and two of her pups—a male, ‘Wiggins’ (pup P1, b. 22 August 2012), and female, ‘Sponge’ (pup P2, b. 6 July 2025).

The seal group at the time of the birth of pup P1 consisted of three adult females (mother ‘Victoria’ (M, aged 10), ‘Franny’ (F2, aged 23) and ‘Fergie’ (F3, aged 10)), two yearlings (male ‘Ollie’ and female ‘Pixie’), and a juvenile female hooded seal (*Cystophora cristata*) ‘Eve’. F3 had a miscarriage in 2009 and has not had another pregnancy since. F2 had a stillbirth on 13 August 2012, a week before M, then aged 10, gave birth at 01:43 on 22 August 2012 to her third live pup (P1). M had previously experienced stillbirths in 2006 and 2008, but had successfully reared male pups ‘Gin’ in 2007 and ‘Sealo’ in 2011. The adult male ‘Sam’, who had sired all the pups born since 2006, died after F2 and M became pregnant, and hence there was no adult male in the group during observations of M and P1.

There was, therefore, no breeding male in the group until Ollie matured, with his first pup being born to Pixie in 2018. M gave birth in July 2021 to a female pup which died four days later from a congenital condition, and M strenuously tried to defend the dying pup from being removed by the Natureland personnel. When she gave birth to P2 in July 2025, the seal group consisted of M, F3, Ollie, Pixie, female ‘Scarlet’ aged 5 years, and male ‘Albie’ aged 2 years. Because M showed such intense defensive guarding of pup P2 against the other seals, the pool was temporarily divided into two parts by a net fence, leaving M and P2 alone in the island pool section (Figure 1), while the surrounding edge of the island pool was closed to viewers. P2 was the only pup born to the group in 2025, owing to contraception intervention by the zoo management.

### 2.2. Behaviour Recording

A closed-circuit television (CCTV) recording system was initially set up in August 2012 in the hope of capturing M’s imminent birth. The four overhead IR cameras of the CCTV unit (STORAGE OPTIONS DIY Home CCTV kit) were attached to the roof of the main zoo building overlooking the pool (Figure 1); the DVR and monitor were placed in the main zoo building (Figure 1). The CCTV recording data was in compressed format, enabling 50 days of DVR recording, saved daily on a series of memory sticks.

The birth of P1 and the 16 min aftermath on the birth beach were successfully captured starting at 01:43 on 22 August 2012 [3]; the CCTV recorder was then left running to capture subsequent behaviour opportunistically until P1’s postnatal day 16, after which time there was no further CCTV recording available. However, on postnatal day 10, it was noticed that the CCTV cameras were not optimally placed for capturing the areas—island pool and birth beach—most frequented by M and P1; the camera positions and angles were then adjusted on 1 September 2012 for improved capture of those areas. Nevertheless, there were always ‘blind’ areas outside the CCTV cameras view when either mother or pup were out of sight or too distant for behaviour description; the CCTV recordings used for the present analysis of M and P1 were limited to the main pool in the early mornings and to the island and the birth beach, where suckling behaviour was frequently captured.

The resulting compressed recordings were viewed using “Playback” software version 2.3.0.4. Snapshots were taken of the recording by the observer each time a noted behaviour (see Section 3.3) was observed; the snapshots gave the time, to the nearest second, of each behaviour.

Additional hand-held video camera recordings of M and P1 were made during two visits by the authors on days 2–4 (using a PANASONIC camcorder with an SD card) and days 7–10 (using a PANASONIC camcorder with mini DV tapes). Video was taken in 5 min segments or longer continuous sessions, depending on the behaviour occurring. One author (S.C.W.) learned of P2’s birth in July 2025 while discussing the retrospective analyses of M’s and P1’s behaviours with Zoo personnel, and was then able to make a visit to observe the behaviour of M and P2 on postnatal days 19–22, i.e., at a later stage of lactation than the earlier observations of P1. Video clips of P2 with M in the island pool area were taken (with a PANASONIC *Lumix*) only while they were in clear view of the observer, whose position was limited to one corner of the island pool (Figure 1). The author (S.C.W.) was invited to observe P2’s release to the sea in January 2026.

### 2.3. Behaviours Described

The behaviours described for M and P1 in the present narrative are the same as those previously variously described quantitatively for mother–pup pairs in the wild [3,5,6,7,8,10] (Table 1). The behaviours described for M and P2 from the hand-held video are listed separately (Table 2) because they were analysed separately from the CCTV data.

Behaviour in the water was termed ‘idling’ when M and P were moving slowly and not ‘travelling’ rapidly around the pool, and ‘static’ when there was little directional movement. Behaviours were classed as ‘underwater’ when at least their faces and muzzles were submerged. The parts of the body that the mother and pups targeted during nosing contacts, the degree of closeness of body contact between the mother and pups, and the orientation and behaviour of the pups while following their mother are described.

**Figure 2:**
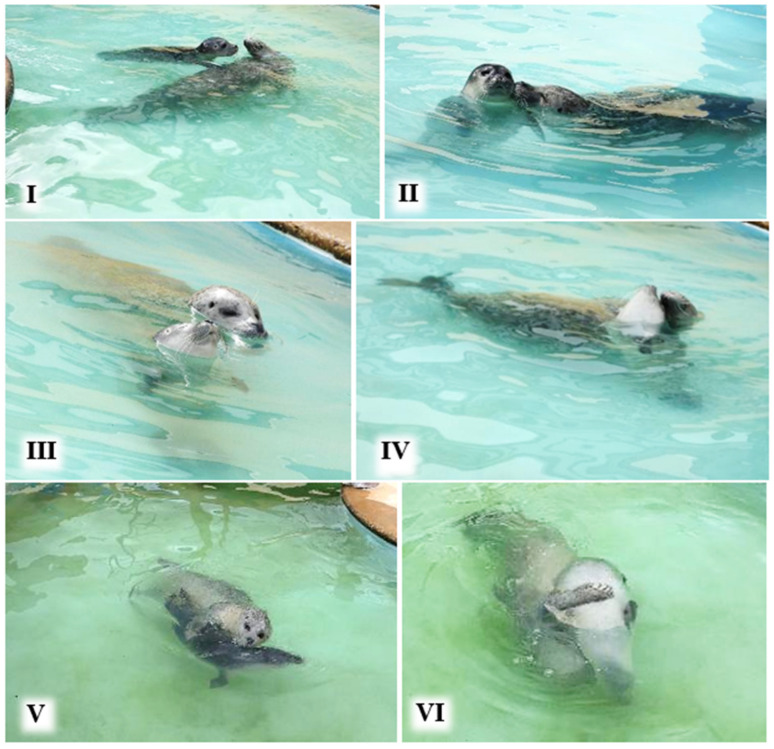
Examples of body ‘static contact’ levels I–VI between M and P2 (see Table 3).

The estimated amount of P2’s body surface area in contact with M during static contact when idling in the water was assigned to a category I–VI, based on similar categories for the estimated amount of body surface area in contact during grey seal juvenile social interactions [31] (Table 3; Figure 2). The contact categories range from fore-flipper touch (level I) to most of P2’s body in contact with M (level VI).

### 2.4. Data Processing and Semi-Quantitative Analyses

#### 2.4.1. CCTV Footage for M and P1

The CCTV footage used in the analysis was the first 30 min from the start of daylight when the mother and pup were present and visible in the main pool. A ‘clip’ was defined as a section of footage of M and P1 from their appearance in the main pool to their disappearance from sight, and a set of snapshots was taken of each behaviour occurrence in each clip. A total of 102 clips (total time 49.7 min, av. duration 28.3 s, S.D. 10.1 s) were recorded during postnatal days 1–7 (‘week 1’) and 114 clips (total time 92.4 min, av. duration 48.7 s, S.D. 4.5 s) during days 11–16 (‘week 2’). The occurrence of each behaviour in each clip was recorded in a spreadsheet, together with a verbal description and reference to the saved snapshots. The number of clips in which each behaviour occurred was used as a measure of frequency of occurrence.

M’s and P1’s approaching and leaving one another in the water were analysed using the percentage of approaches and percentage of leavings (*% App* − *% L*) due to M or P1; this measure affords an estimate of responsibility of mother and pup for maintaining proximity [5,6,32]. A chi-squared (χ^2^) test was used to compare the frequency between the two time periods of M and P1 leaving or following one another.

#### 2.4.2. Video Footage for M and P2

Video recordings of M and P2 were made during 3 h in the early afternoon of postnatal days 19–22. From the video clips of M and P2 in the water (*N* = 74; total time 101.1 min, av. duration 82 s, S.D. 6.7 s), snapshots were taken each time the mother and pup’s positions changed. Each snapshot was then assigned to one of the behaviour categories listed in Table 3.

## 3. Results

The mother–pup behaviour observed fell naturally into three main categories: (1) behaviour as the pair moved in the water around the pool (‘travelling’, ‘idling’ or ‘static’), (2) behaviour as the pup or both mother and pup rested on a beach, and (3) behaviour related to suckling.

### 3.1. Behaviours Observed in Water—M and P1

**Figure 3:**
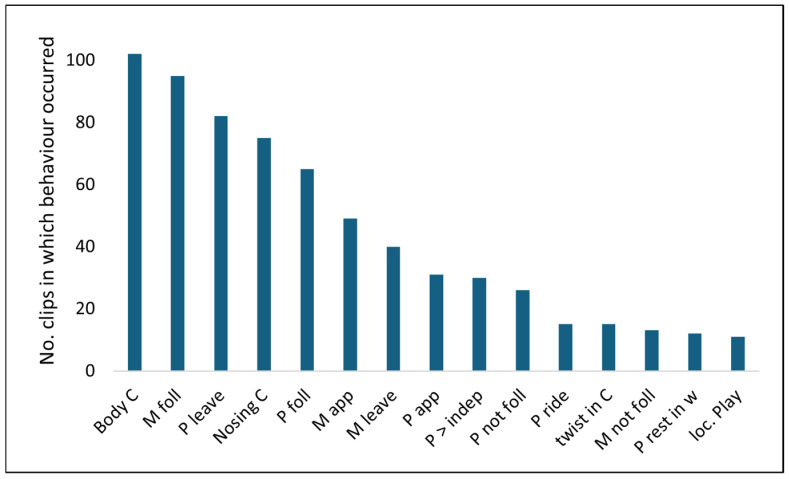
Aquatic behaviours of M and P1 in the water in descending order of occurrence (no. clips in which the behaviour occurred; CCTV footage—11 days recorded during days 1–16; *N* = 216 total no. clips in weeks 1 and 2). P = P1; C = contact; foll = follow; app = approach; >indep = swims independently; w = water; loc. = locomotor.

During the period in the early mornings when both M and P1 were recorded in the water, the most frequently occurring behaviours recorded on the CCTV were body contact between M and P1, M following P1 as he moved away from her, nosing contacts between them, and P1 following M (Figure 3).

#### Static Body Contact Behaviour in the Water—M and P1

**Figure 4:**
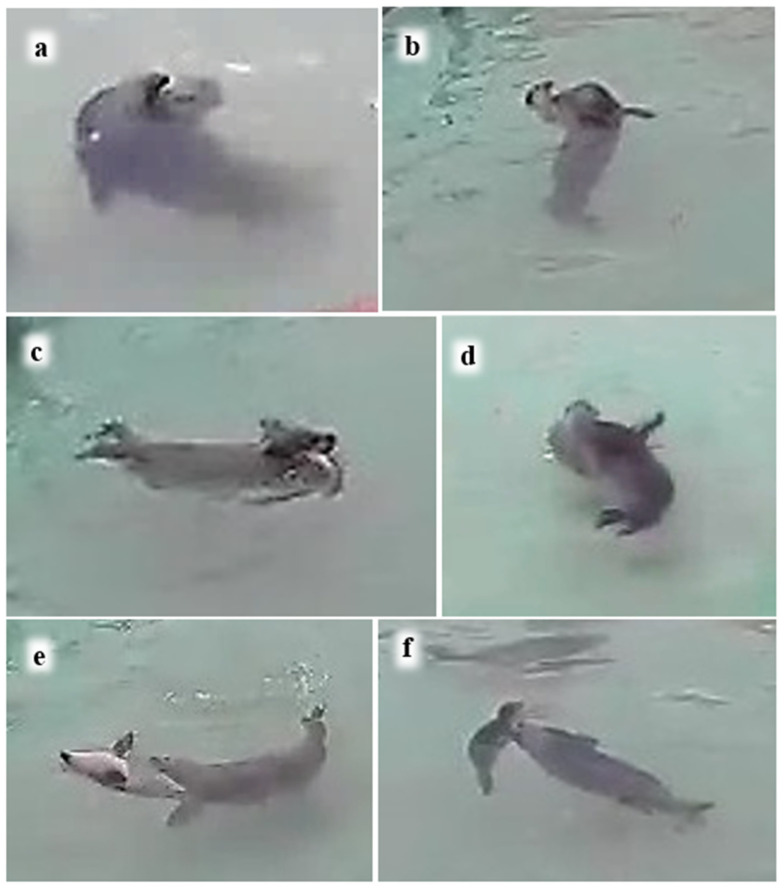
Examples of static contact patterns in the water between M and P1 (CCTV recordings). (**a**,**b**) M stretches backwards over P1, contacting him with the back of her head/neck; (**c**) M leans her head and chin over P1; (**d**) M and P1 twist in contact; (**e**) M leans her chin against P1’s rear venter while pup is supine; and (**f**) M propels P1 by pushing his side-of-neck.

**Figure 5:**
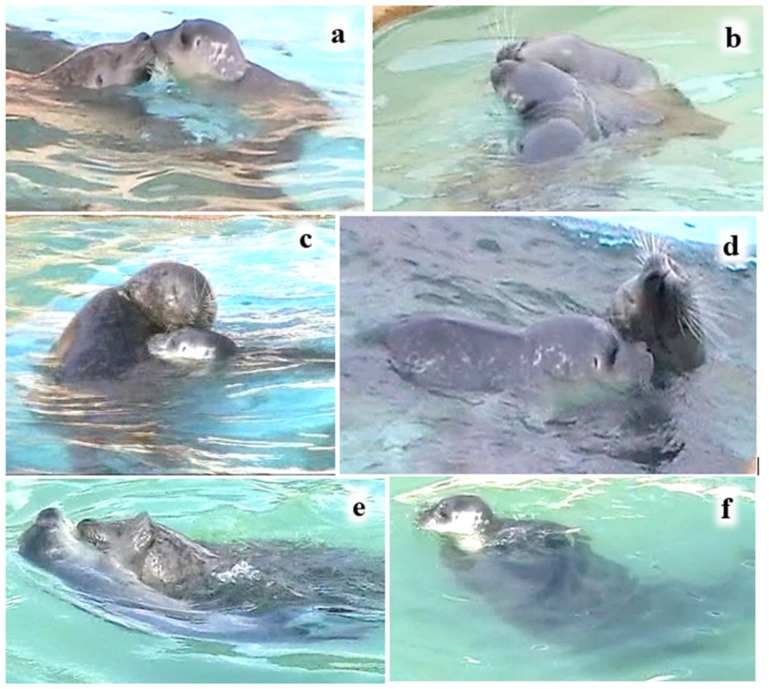
M’s and P1’s nosing and body contact patterns in the water (video record). (**a**) Nose-to-nose, with M’s and P1’s eyes closed; (**b**) nose-to-nose, with M stretching back to P1; (**c**) M stretches her head round to nose P1’s top-of-head, P’s nose is to M’s shoulder, and both have eyes closed; (**d**) heads are in contact, M stretching back to P1, M’s eyes closed, and pup’s eyes nearly closed; (**e**) M initiating venter-to-venter contact, P1 is supine, P’s fore-flipper is on M’s head, and M’s eyes are closed; (**f**) M holding P1 in her fore-flippers, with P’s eyes nearly closed.

Static body contact was recorded with similar frequency in week 1 (occurring in 48 of 102 clips) as in week 2 (in 54 of 114 clips). It was most often initiated by the mother (Figure 4 and Figure 5). M’s eyes were typically closed and P1’s eyes were either closed or partially closed. A relaxed body tone was evident in both mother and pup, as evidenced by the loose ‘curvy’ appearance of the mother’s whole body (Figure 4).

### 3.2. Body Surface Area of P2 in Static Contact with M in the Water

Snapshots of static contact behaviours (total *N* = 122) indicated that the most frequent body contact levels (Figure 2, Table 3) were III–V, with level IV, equivalent to about one third of the pup’s body, recorded most often (Table 4). Snapshots of contact behaviours recorded included M leaning or stretching her head backwards over P2 (*N* = 48), P2 stretching back to M (*N* = 3), the pup leaning over or against its mother (*N* = 68), and the two mutually leaning their heads round each other (*N* = 6). M held P2 between her flippers with levels VI (*N* = 9), III (*N* = 2) and V (*N* = 2) contact. M’s eyes were closed almost all the time (*N* = 54), with her eyes half open (*N* = 4) or fully open (*N* = 1) less often. However, P2’s eyes were closed relatively less often (*N* = 31), and her eyes were half open (*N* = 14) and fully open (*N* = 17) more often. P2’s eyes were closed approximately equally often during contact levels III, IV, V and VI (*N* = 7, 7, 9 and 6, respectively), and rarely (*N* = 2) during minimal contact.

### 3.3. Body Nosing Contact Between M and Her Pups While Idling in the Water

Most of the nosing contacts between the mother and both pups when they were idling in the water were nose-to-nose (*N* = 32), to the head and neck area (*N* = 49), to the face and throat (*N* = 11) and to the hind-flippers (*N* = 15).

**Figure 6:**
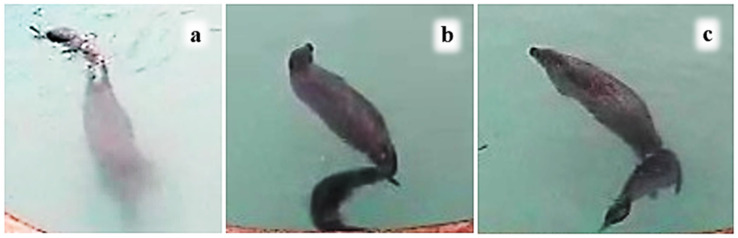
(**a**–**c**) P1 following M making nose-to-hind-flipper contact, often just beneath the surface (CCTV record).

All the nose-to-nose contacts between M and P1—and all unilateral nosing contacts by M to either pup—occurred above the surface. However, although P1 nosed M above the surface most often, he also made some unilateral nosing contacts underwater (Table 5). Hind-flipper nosing by P1 occurred when he was following her as she moved very slowly in the main pool (Figure 6).

**Figure 7:**
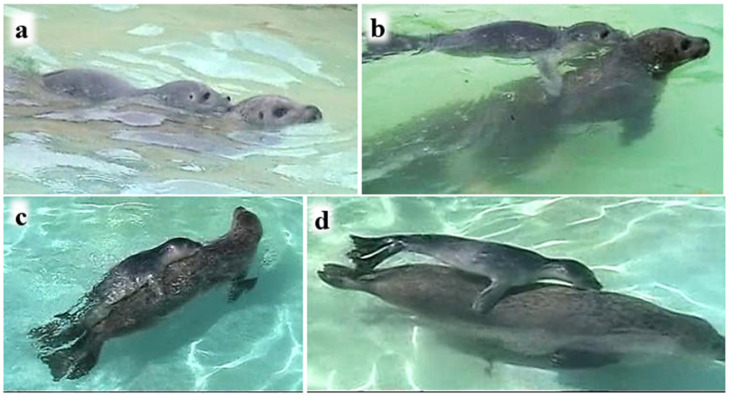
P1 riding with nose contact above the surface, with mother’s back-of-head/neck (**a**,**b**) or shoulder (**c**,**d**); pup riding in close body contact (**a**,**c**), and in loose contact (**b**,**d**).

**Figure 8:**
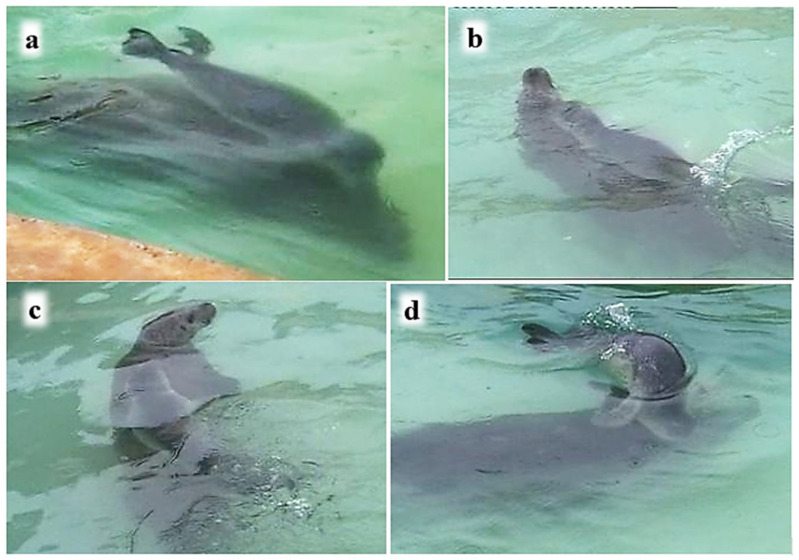
(**a**–**d**) P1 making nosing contacts underwater to mother’s back-of-head region, mainly in the context of riding.

However, when P1 was riding on M with his nose to M’s back-of-head or shoulder, the pup’s nosing contact occurred either above the surface (Figure 7) or underwater (Figure 8). Riding involved either close contact between the pup’s venter and the mother’s back (Figure 7a,c) or light contact, sometimes with just the pup’s fore-flippers (Figure 7b,d).

M sometimes stretched her head back to nose P1 (*N* = 12, Figure 5b,c), while he rarely did so to M (*N* = 1). M occasionally propelled P1 along by pushing his back-of-head (*N* = 2) and once pushed him along slowly by a prolonged nose-to-nose contact. The frequency of all nosing contacts per CCTV clip was similar in week 1 (0.32) and week 2 (0.36).

The nosing contacts recorded for M and P2 (*N* = 38) while they were idling in the water were almost all at the surface (*N* = 37), and almost all to the muzzle, face, head and neck (*N* = 35). They nosed each other equally often, although M initiated seven of eight nose-to-nose contacts. The frequency of all nosing contacts per video clip in the water (*N* = 94) was 0.40.

### 3.4. Movement Dynamics in the Water

#### 3.4.1. Following, Leaving and Approaching in the Water by M and P1 (CCTV Recordings)

**Figure 9:**
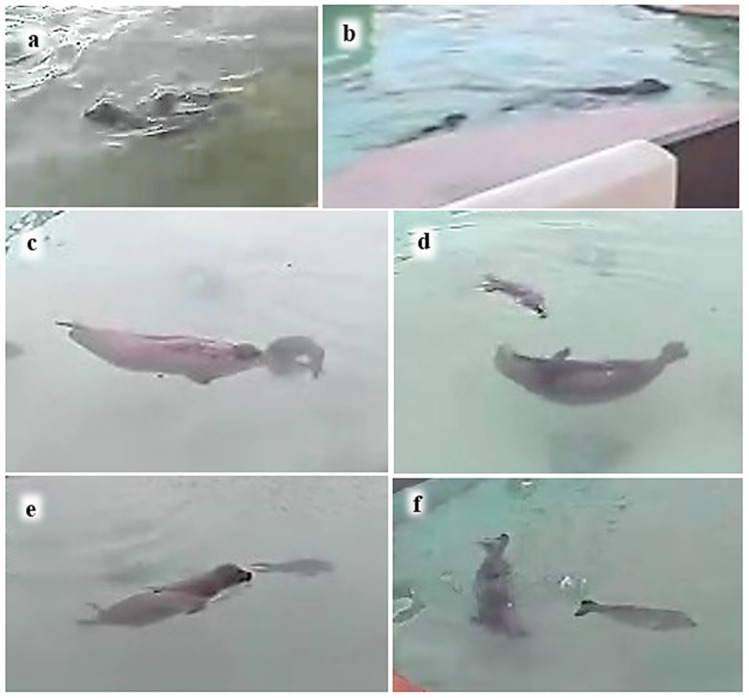
Examples of movement dynamics between M and P1 in the water (CCTV recordings). (**a**) P1 follows M, orienting to her back-of-head; (**b**) P1 follows M’s hindquarters; (**c**) M approaches P1; (**d**) P1 approaches M; (**e**) M follows P1; and (**f**) P1 leaves his mother.

P1 and M following each other were characteristic behaviours when they were idling around the main pool (Figure 9a,b,e), frequently approaching one another (Figure 9c,d). The *% app* − *% L* measures due to P1 in weeks 1 and 2 were −7.8% and −39.0%, respectively; i.e., P1 always swam away from M more often than he approached her (Table A1).

P1 also left M (Figure 9f) more often than M left him (54.5% of leavings due to P1 in week 1 and 77.3% in week 2 (*p* = 0.008, χ^2^ test)). When P1 swam away from M, she responded by following him more often (93% of occasions) in week 1 (*N* = 30) than in week 2 (21%, *N* = 52) (*p* = 0.038, χ^2^ test). On nine occasions when the pup left his mother, he then promptly returned to her side. However, P1 showed an improved following response to M when she moved away, following her on 21% of occasions in week 1 (*N* = 23), but on 60% in week 2 (*N* = 15) (*p* = 0.039, χ^2^ test) (Table A1).

M and P1 moving away from each other was preceded by body contact in 55% of 38 instances of mother moving away from the pup and in 67% of 82 instances of the pup moving away. However, in four instances (on day 4) when P1 was sleeping in the water, M approached to check him and left again without making contact.

#### 3.4.2. Following Behaviour in the Water by P2

**Figure 10:**
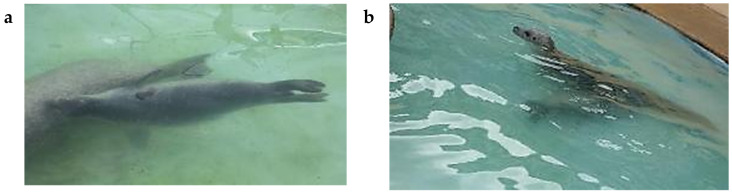
P2 contacting M while swimming underwater. (**a**) P2 noses M’s flank while swimming side-by-side underwater; (**b**) P2 dives underneath M, in contact with her venter.

P2 always followed M when she initiated rapid swimming around the island pool. About half the records of nosing contact by P2 occurred underwater (Table 6; see also Table 2). P2 frequently rode on M’s back, mostly when the pair were at the surface, although occasionally underwater (Table 6). When M swam rapidly round the island pool with P2 following, P2 sometimes swam side-by-side with M, mostly underwater (Table 6), and twice with underwater nosing contact (Figure 10a).

P2 sometimes dived underneath M, making body contact with M’s venter, most often with her abdomen or chest (Figure 10b), but sometimes with her hind quarters or throat.

### 3.5. Resting on Haul-Out Platform—M and P1

**Figure 11:**
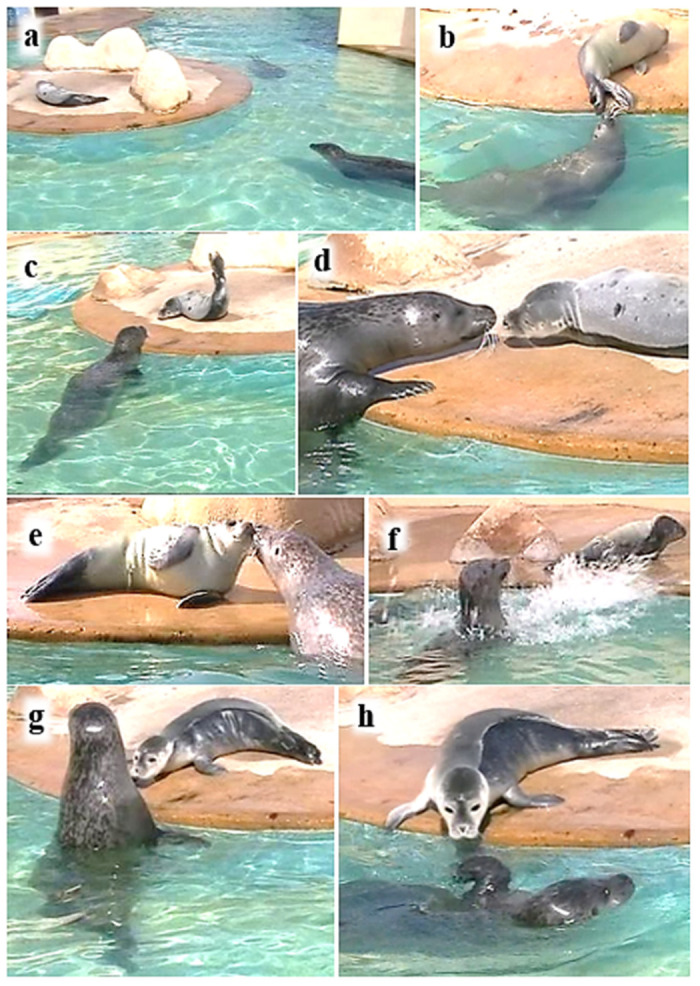
P1 resting alone on island—examples of mother (**a**) visually checking pup, (**b**) nosing sleeping pup’s hind-flippers, (**c**) approaching pup closely and pup responding, (**d**) touching whiskers, (**e**) nose-to-nose contact, (**f**) splashing pup to gain his attention, (**g**) mother waiting while pup stretches forward to nose her neck, (**h**) mother swimming closely past, waiting for pup to join her in the water (**b**, **e** and **f** from video taken on 24 August (day 3), others taken on 31 August (day 10)).

While P1 rested alone on days 3 and 9, M checked on him constantly from 1 to 3 adult body lengths (ABLs) away (Figure 11a). When there was no disturbance, she approached him frequently to ~1 pup body length (PBL), averaging nearly once a minute on day 3 and nearly once every two minutes on day 9, making nosing contact nearly every time she approached (Table 7; see also Table 1). However, during a 10 min visitor presentation with the loudspeaker on day 3, M returned to check on the pup six times but did not make any contact with him.

The hind-flippers of the sleeping P1 were often most accessible to M (Figure 11b) and most often nosed by M when checking on him. However, she only occasionally made body contact with him unless she was trying to waken him—she would then rouse him by leaning over him and making nosing contact. When she had his attention, she would approach closely (Figure 11c), make whisker contact (Figure 11d), or engage him in close nose-to-nose contact (Figure 11e). On day 3 she attempted to gain the sleeping P1’s attention by nosing and nudging, and by splashing at him on six occasions (Figure 11f). After the sixth splashing attempt, M leaped onto the island and nosed the pup, although he continued to sleep. On day 9, after M made whisker and light nose-to-nose contact, P1 moved towards the island’s edge, watching M (Figure 11g), and finally joined her in the water (Figure 11h).

**Figure 12:**
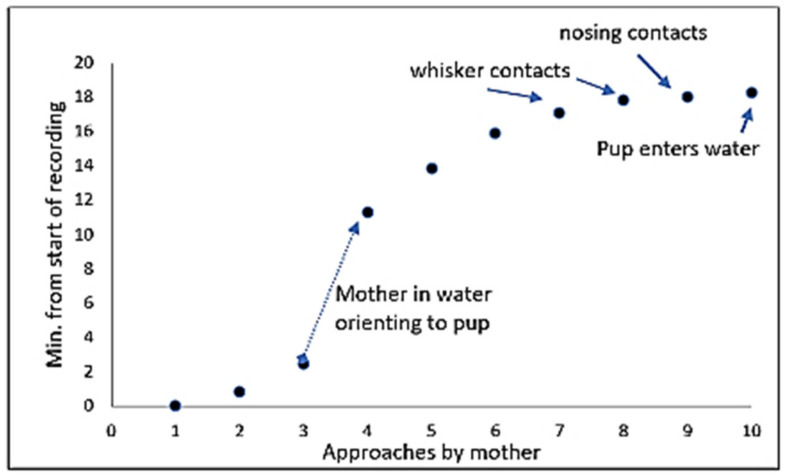
Timing of 10 approaches by M while P1 was resting on the island (postnatal day 10) until he joined his mother in the water (minutes from start of video recording).

On day 10, a continuous video of M and P1 for 25 min documented the mother’s tactics on that occasion to coax her sleeping pup into the water (Figure 12). Between minutes 2 and 11, M hovered in the water, orienting to the pup from 1–3 ABL. At min 16 she made nosing contact with him, waking him; he then engaged with her in mutual whisker contact. Thereafter, M made more frequent approaches, engaging him in further whisker and nose-to-nose contact, then nosed his head and face, and finally P1 nosed her neck in response. M then swam very slowly, eyes closed, beside the edge of the island, and the pup dived into the water, heading directly to M. M responded by holding him between her fore-flippers with her chin leaning on his head (Figure 4f). For the remaining 5 min of the video recording, M and P1 travelled together in the water.

M and P1 were observed to be hauled out and resting together on only one occasion, on the island on day 3. The video record suggests minimal body surface in mutual contact (Figure A2). However, the body contact between M and P2 included the mother leaning against the pup, mutual leaning by mother and pup, and the pup leaning against the mother (Figure A3).

The nosing contacts when either or both M or P2 were hauled out were mostly to the face, head, throat and neck regions (*N* = 35), with the remainder (*N* = 19) to the shoulder, flank, or fore-flipper. Mutual nose-to-nose contact occurred less often (*N* = 7). Of these 35 nosing contacts, 29 were followed by suckling.

### 3.6. M and P2 Orienting Towards One Another

**Figure A1:**
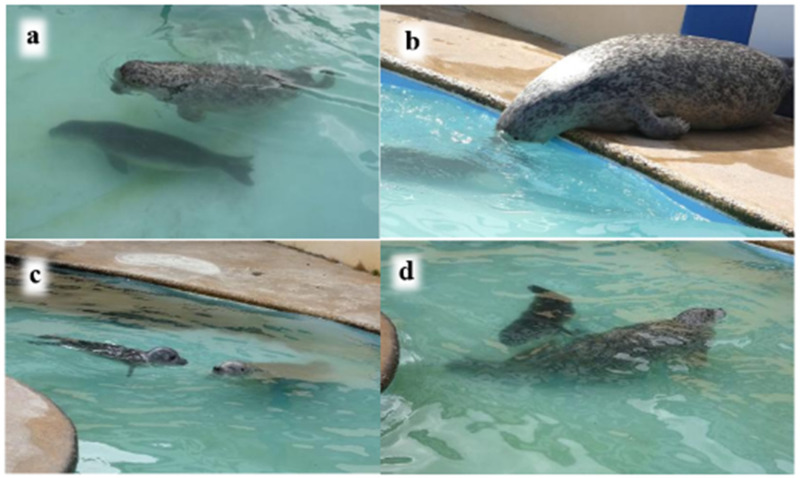
M and P2 orienting towards one another. (**a**) M hovers over P2, (**b**) M dips head underwater to watch P2, and (**c**,**d**) P2 orients towards M at surface and underwater.

**Figure A2:**
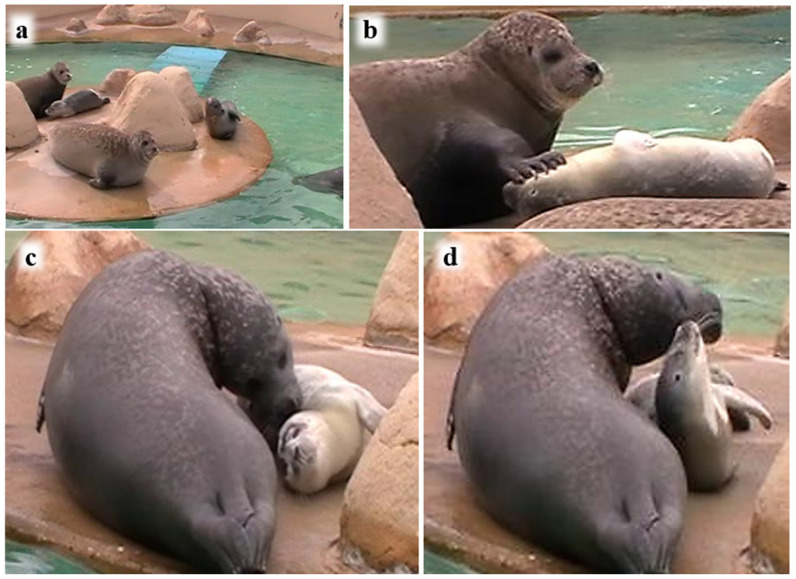
Nosing and body contact while hauled out and resting together. (**a**) M and P1 resting together on the island (juvenile and adult female F2 also resting on the island; “birth beach” in background), (**b**) M lays fore-flipper on sleeping P1, (**c**) M noses P1’s back-of-head, and (**d**) P1 reciprocates by nosing M’s top-of-head.

**Figure A3:**
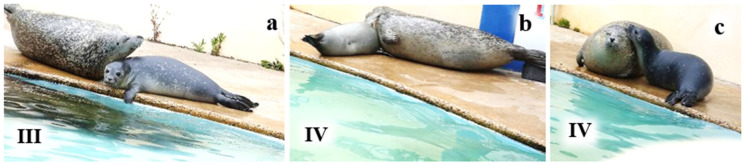
Body contact between mother and pup P2 when hauled out, with estimated body contact levels III and IV. (**a**) Mother leans against pup, (**b**) mutual leaning (pre-suckling), (**c**) pup leans against mother (post-suckling). Mother’s eyes are closed in (**a**,**c**).

M and P2 often oriented towards each other (*N* = 31, M to P2; *N* = 17 in the water; Figure A1a) (*N*= 48 for P2 to M; *N* = 30 in the water; Figure A1d). When M was hauled out while P2 was still in the water, she dipped her head in the water on four occasions (Figure A1b), while P2 did so on one occasion when she was hauled out while M was underwater.

### 3.7. Behaviour in Relation to Suckling

Most of the observed 29 suckling bouts by P1 occurred on the birth beach (Table A2). The first attempted suckling occurred on day 1, just over 5 h after the birth (at 01:40) and lasted only 43 s. Five further attempts were made up to 03:50 on the next morning, with the first substantial suckling—apparently lasting more than 19 min—not occurring until nearly 15 h after the birth. From day 2 on, the length of uninterrupted suckling bouts varied between 3.5 and 11 min (Table A2).

**Figure A4:**
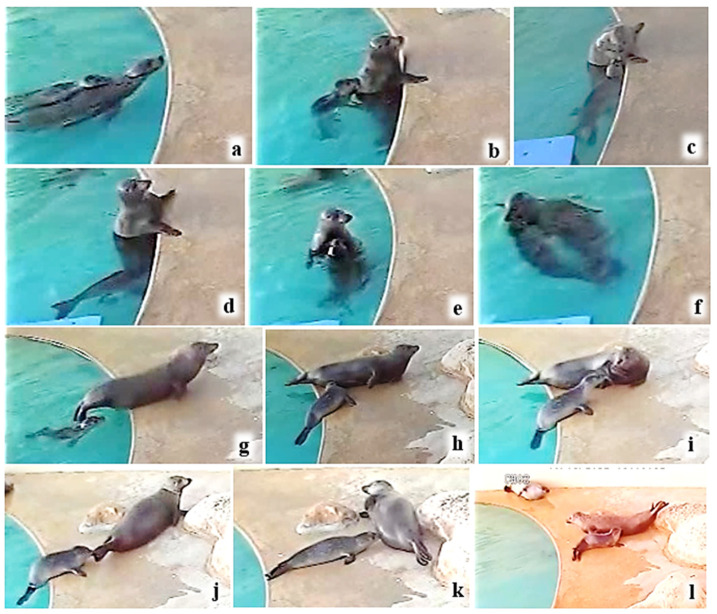
Example of suckling sequence beginning with M leading pup P1 to a haul-out platform—the ‘birth beach’ (snapshots from CCTV recording, 5 September 2012, day 15). (**a**) P rides as M approaches haul-out (06:03:44); (**b**) pup noses M’s shoulder; (**c**) M turns and makes nose-to-nose contact with P; (**d**) P noses M’s hindquarters underwater; (**e**) P noses M’s chest; (**f**) M and P dive back into water together in head-to-head contact; (**g**) P follows M’s hindquarters as she hauls out; (**h**) P hauls out after M, suckles immediately (06:04:43), (**i**)P detaches and makes nose-to-nose contact with M (06:07:58); (**j**) M hauls out further, P follows, nosing her hind-flippers; (**k**) P suckles again (06:08:43); (**l**) M terminates suckling by raising her hindquarters and shifting position (06:09:32); 2nd seal is hauled out at the spot where the birth occurred.

Nineteen suckling bouts (where the start of suckling was recorded) were preceded by M leading P1 to haul-out on the beach (Figure A4). On 11 of the 16 occasions when suckling was observed occurring directly after M and P1 hauled out together, M presented immediately with no nosing contact preamble, and P1 went directly to the nipples. When pre-suckling nosing contacts by P1 to M occurred, the pup solicited suckling by nosing M’s muzzle, chest, shoulder, back, and—on day 16—leaning its chin over the mother’s body and patting her several times (Table 8).

When M and P1were already hauled out (or stranded on the floor of the drained pool), there were no nosing contacts leading to suckling on 3/7 occasions. However, on all other occasions recorded suckling was preceded by reciprocal nosing (Table 8).

**Figure A5:**
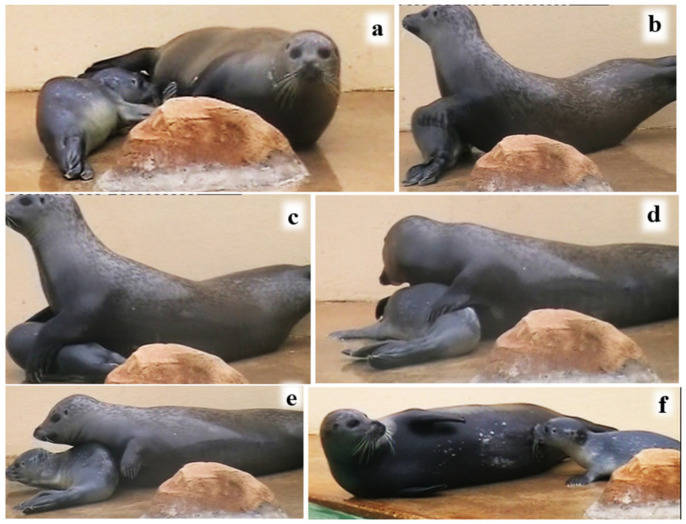
Suckling interrupted by mother responding to loud voices (snapshots from hand-held video recording, 30 August 2012—day 9). (**a**) Mother suckles P1; (**b**) startled by loud voices, terminates suckling by raising hind quarters; (**c**) covers pup with both fore-flippers, leaning over pup; (**d**) relaxes, checking pup; (**e**) releases pup from fore-flippers; (**f**) allows pup to suckle again.

The suckling bouts were most often terminated by M (19 of 27 occasions recorded; Table A2) either moving her hind quarters away from the pup or by diving into the water. In eleven cases, M was distracted, either by one of the other two adult females approaching them (nine times) or by loud voices (twice; Figure A5). However, on four occasions suckling was resumed after several minutes (days 7 and 9) or after about 3 h (days 13 and 15; Table A2).

M was recorded presenting to P2 on five occasions (days 21 and 22), although a suckling bout was only recorded twice, both times immediately preceded by mutual body contact. The pre-suckling nosing contacts by P2 were all to M’s face, head and neck. Following each suckling occasion, P2 initiated return to the water.

### 3.8. Pup Weaning and Release to Sea

P1 was weaned naturally at 18.4 kg on approximately 16 September 2012 (postnatal day 26), fed by hand from day 27, and released (with a hind-flipper tag) on 10 January 2013, at 20 weeks of age and 26.5 kg (Natureland personnel, pers. comm.). P2 was weaned naturally between 6 and 10 August 2025 (days 32–37), with suckling bouts declining from day 32. Her weaning weight was 23 kg, and she was removed from the seal group and fed by hand from day 39 and released (with a hind-flipper tag) on 29 January 2026, at 29 weeks of age (Natureland personnel, pers. comm.). She was released in the company of a rehabilitated male pup (‘Rice’) that stranded on 13 August 2025 and with whom P2 had been co-habiting in a group of rehabilitating pups for several weeks.

**Figure A6:**
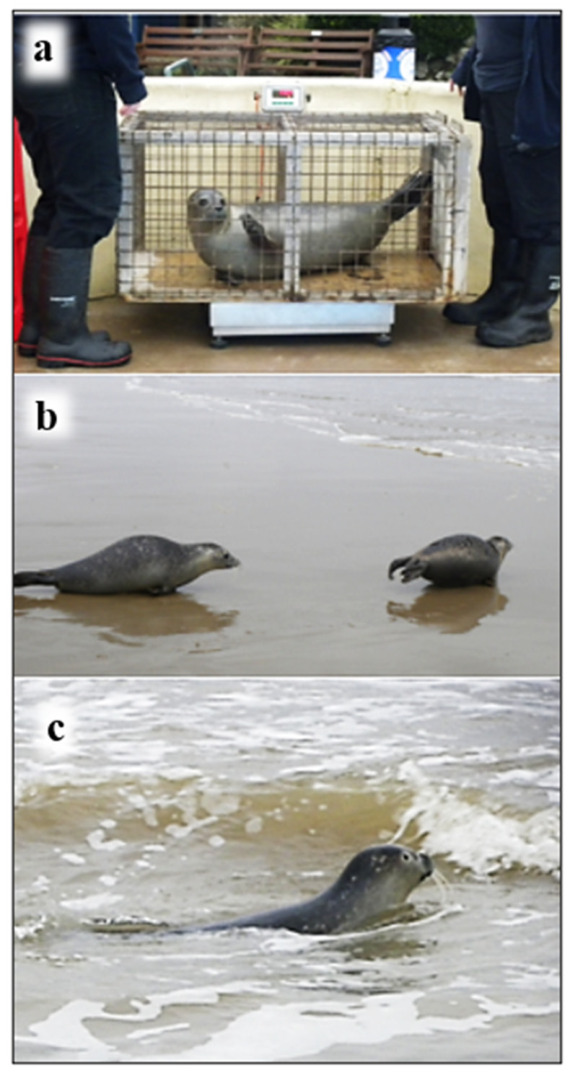
Captive-born pup P2 (‘Sponge’, 29 weeks of age) being released to sea with rehabilitated pup ‘Rice’ (29 January 2026). (**a**) P2 being weighed moments before release, (**b**) Rice heads rapidly into the sea followed by P2, (**c**) P2 strenuously following Rice (out of shot) as he heads further out to sea.

P2 followed Rice closely as they entered the wavelet zone at Skegness (53.149° N, 0.351° E) (Figure A6). The straight distance to the nearest known seal haul-out—on an offshore sandbank at Gibraltar Point—is ~6 km southwards.

## 4. Discussion

Our central hypothesis motivating this study was that the captive harbour seal mother Victoria would display all the natural behaviours of wild mothers caring for their dependent pups moving in and out of the water at their pupping sites [10], while displaying flexible behavioural adjustments according to circumstances [3]. We also wanted to explore whether these captive observations might lead to new insights into harbour seal mother–pup behaviour as well as considerations of their overall welfare and potential positive outcomes of captive breeding of harbour seals.

### 4.1. Behaviours Observed in Captive and Wild Mother–Pup Pairs

The captive mother Victoria (M) and her pups (P1 and P2) displayed all the most frequently recorded active behaviours of wild harbour seal mother–pup pairs from field observations. Body contact between M and P1 occurred in 47% of CCTV clips, which is similar to ~40–60% of time it occurred between wild mother–pup pairs in an intertidal zone, where their behaviour in the water was described from an elevated observation position [5]. The frequency of occurrence of nosing contacts between M and P1 in the water was 35% of CCTV clips, of which 13% occurred underwater and 22% at the surface—similar to 25% of time at the surface recorded by a wild study (where underwater observations were not possible) [10].

The most frequent body contact levels, III and IV, between M and P2 in the water are similar to the most frequently recorded contact level, III, in grey seal mother–pup pairs—who spend most of their time on dry land during the suckling period—and juveniles at the water’s edge [30]. However, the greater body contact levels V and VI displayed by M and P2 in the water (pup riding or mother holding pup) were only equalled by the grey seal mother–pup pairs on the relatively rare occasions when they entered the water together [31].

The predilection of both harbour and grey seals for body contact in the water, or at the water’s edge while still wet, is thought to relate to the increased sensitivity of the *C low-threshold mechanoreceptors* (CLTMs) of hairy skin when wet [33]. During gentle touch, the afferent nerves of CLTMs transmit signals to the emotional systems of the frontal lobe, resulting in stress reduction [33,34]. Gentle or light contact (levels I and II, e.g., fore-flipper touch) equates to a *pleasant touch*, while contact levels III–VI equate to *deep pressure* providing a *subjective pleasant sensation* [35], likely explaining the observed relaxed body tone and closed eyes of M and both P1 and P2, which may be interpreted as indicating a state of behavioural focus on the sensations of mutual body contact and positive emotion in both mother and pup. The close contact observed between them would be expected to result in increased oxytocin, dopamine and rewarding endorphin and serotonergic systems [36,37,38].

Observer visual perception of ‘relaxed body tone’ has been used in description of mother–pup behaviour in grey seals [31], in the ‘relaxed open mouth’ (ROM) favouring playful contact in captive South American sea lions, *Otaria flavescens* [39], while half-closed eyes have been considered indicative of relaxed emotional state in domestic cows, *Bos taurus* [40]. Studies of domestic cattle have correlated observer reports of ‘relaxed/calm’ behaviours from Qualitative Behaviour Analysis (QBA) with quantitative records of ‘social licking’, thereby demonstrating the descriptive validity of observer visual reading of mammal body language [41].

### 4.2. Flexible Maternal Behaviour Adjustments Displayed by Mother Victoria (M)

Observations in the wild have shown that harbour seal mothers of young pups usually remain constantly close to them and do not leave them resting alone at a haul-out site until ~10–14 days post-partum, when a mother may leave her pup at a haul-out site while she leaves to forage in order to sustain lactation [42]. However, M was observed leaving P1 sleeping alone on the island during days 3 and 9. She also sometimes swam away from P1 in the water during the first week, sometimes while he was sleeping. One possible interpretation of this laissez-faire behaviour might be that M was confident that there was no danger to her pup from the other seals or human carers. Nevertheless, she watched him constantly from a few metres away while he was sleeping, frequently approaching to check him closely. Her clear signals to P1 when he was resting on the island (nosing and body contact, splashing at him, waiting for his response) suggest she had a cognitive awareness of his responsiveness. Splashing to get their pup’s attention has also been employed by wild seal mothers observed in Shetland and the west of Scotland [5], although it has not been noted elsewhere, suggesting it may be an ad hoc signal devised by individual mothers.

In the wild, pups and their mothers spend up to about 60% of their time in the water [4]. Much of this time may be while the nursery haul-out sites are flooded at high tide. However, in a captive situation the haul-out ‘beaches’ are always available, and yet M and both her pups spent most of the daytime in the water, hauling out only to suckle and returning to the water immediately after suckling. This suggests a psychological need of both mother and pups to spend time in the water, a suggestion substantiated by two recent studies of rehabilitating orphan harbour seal pups, in whom the stress hormone cortisol was elevated while they were without free access to water [43,44]. Mother–pup pairs observed in the wild during daytime also appear to spend more time idling, swimming and following in the shallow water around the haul-out site than they spend resting onshore [5,10]. With P2, M often swam rapidly, clockwise round the island, closely followed by the pup; this could possibly be interpreted as ‘pattern’ swimming [45] occurring as a substitute for the prolonged swimming a mother in the wild would make on a foraging trip at this late stage in lactation, sometimes accompanied by her pup [46,47].

### 4.3. Trends Observed in Mother–Pup Behaviour Frequencies from Early to Late Lactation

Some continuities and changes in the frequency of occurrence of behaviours were observed for M and the pups, which are consistent with some previous observations of wild pups. The frequency of both static body contact and nosing contacts in the water for M with P1 remained constant between week 1 and week 2, appeared at least as high for M and P2 during days 19–22, and both pups were continuing to ride on their mother’s back by the end of observations. This maintaining of mother–pup body contact levels in later lactation was also found in a study of wild mother–pup pairs, although in that study a decline of ~50% in the frequency of nosing contacts was noted from the beginning to the end of the pupping season [30].

P1’s following response to M appeared to improve in the second week, consistent with observations of wild newborn pups, whose following response may be slow to develop [48]. Pup P1 dived away from M more often than the reverse in both the first and second week—as was also the case for wild pups and their mothers throughout the lactation period, while the percentage of approaches due to P2 (27–38%) was also similar to the 32–52% by wild pups [5]. P1, in his second week, displayed an increase in independent swimming but also approached his mother more often.

In summary, the dynamics of a captive mother–pup relationship appear similar to those observed in wild harbour seal mother–pup studies and indicated no overt waning of their mutual bond during week 2 (P1) or by days 19–22 (P2). The strength of the mother–pup bond is likely mediated by the hormone oxytocin [34,49]. For wild pups, the likely continuing oxytocin levels and associated contact behaviours would help to prevent the pup from going astray if it accompanied its mother on a foraging trip [46,47], and would also ensure the reunion of mother and pup in late lactation following a foraging trip where the pup was left behind at the haul-out site [30].

### 4.4. Suckling

M usually initiated suckling by leading P1 from the water directly to the birth beach. Nursing immediately following mother–pup haul-out has been noted in the wild [5,10,11,13]; this was also the pattern for M and P1, lending further evidence to counter the view that mothers and pups lie onshore for as long as possible at low tide to maximise their daily nursing time [50]. Harbour seal pups in the wild can suckle in the water [1,12], although this was not recorded for either P1 or P2.

Lawson and Renouf [8] reported that pups in the wild were increasingly responsible for maintaining nursing, while their mothers often increasingly rebuffed them. Although our study did not include the period immediately preceding weaning (average of 24 days [51]), M was not seen to rebuff either pup, continuing to initiate most nursing throughout our observation period. This observation, made in captivity, is consistent with an earlier field study [5] in which wild mothers continued to initiate nursing until weaning.

The guarding behaviour displayed by M when disturbed by a sudden noise—when she instantly covered P1 with her fore-flippers and body (Figure A5)—has not, to our knowledge, been documented in the wild. M also led P1 back into the water when either of the other two females hauled out beside them, trying to sniff the pup. However, after most instances of interrupted suckling, M and P1 resumed suckling after rehauling out; such resumption of suckling is a natural behaviour in the wild, where mothers lead their pups from one haul-out site to another, either due to a disturbance or the state of the tide [10].

Our observations indicated that M terminated nursing more often than did P1, although on several occasions this was a direct result of disturbance by another seal approaching. A captive grey seal mother also terminated suckling most often, with a disturbance also being suggested as a possible cause [23]. In relatively undisturbed situations, pups or calves have been recorded as ending suckling more often than their mothers among wild harbour seals [5], captive grey seals [22,25,52], and captive walruses [29].

### 4.5. A Hypothesis Arising from This Captive Study of Mother–Pup Behaviour Underwater

In most field study situations, much of the aquatic mother–pup contact behaviour described in this captive study would be difficult to see, since most of their bodies would be underwater even when their heads were mostly at the surface (e.g., Figure 2, Figure 4 and Figure 5).

When a pup in the wild accompanies its mother on foraging trips, much travelling takes place with the pup following and riding on her back, often underwater (Figure 7 and Figure 8). Our captive observations were able to glimpse mother and pup orienting attentively towards one another underwater (Figure 6 and Figure A1), P1 riding on M’s back with his nose to her back-of-head or shoulder (Figure 8), following her with his nose to M’s hind-flippers (Figure 6), and P2 swimming side-by-side with M and with her nose to M’s body (Figure 10). The harbour seal’s back-of-head/neck region is known to be rich in sebaceous glands [53], and the hind-flippers may bear anal gland secretions [54], all of which are likely odoriferous. This raises the question of whether the senses involved in the pups’ underwater nosing may have an olfactory as well as tactile component.

**Figure A7:**
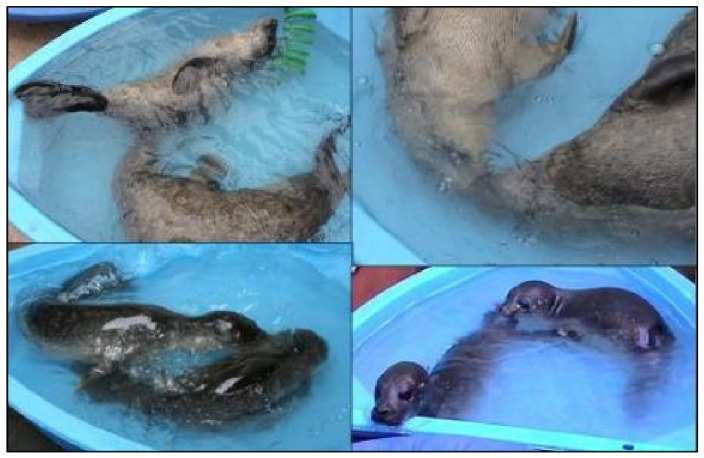
Underwater body nosing between young “orphan” harbour seal pups in rehabilitation.

However, underwater chemoreception would require an active vomeronasal organ (VNO) able to detect volatile, water-insoluble chemicals underwater, as occurs in the Steller sea lion *Eumetopias jubatus* [55]. However, the organ corresponding to the sea lion VNO in adult and subadult harbour seals has been found to secrete acidic mucopolysaccharides, and would therefore not be active in chemoreception [56]. Nevertheless, in our study both pups P1 and P2—but *not* the mother—were observed making underwater body nosing contacts (Table 5 and Table 6). Young orphan pups in rehabilitation also constantly engage in body nosing beneath the water surface [57] (Figure A7). This underwater body nosing by pups suggests the hypothesis that the VNO might function temporarily in young harbour seal pups as a chemoreceptor for detecting body odours underwater, thus facilitating the pup’s underwater riding and following response to its mother. According to this hypothesis, a harbour seal pup VNO would function as a chemoreceptor for the first few weeks post-partum, and would then undergo post-weaning developmental change to become the secretory organ, as described for older seals [56].

### 4.6. Welfare Aspects of Harbour Seal Captive Breeding

The welfare of harbour seals in captivity has frequently been raised, including efforts to provide forms of ‘enrichment’ to try to reduce stereotypical behaviours, such as pattern swimming [45,58,59]. Such pattern swimming by M was not noted during our observations. Even her brief spurts of very rapid underwater swimming around the island pool, always closely followed by P2, did not give the impression of stereotypical pattern swimming. Although the island pool could not provide the space or habitats resembling the harbour seal’s offshore travelling and foraging habitat in the wild [14], it is possible that these swimming spurts of mother closely followed by her pup may have been displaying an element of M leading and chaperoning her pup, as would naturally occur in the wild when a mother leads her pup offshore.

By contrast, the aquatic ‘idling’ behaviour of M and both pups, with their high levels of nosing and body contact with evident positive emotion, and M’s continuously focused and devoted maternal care, closely resemble the very similar mother–pup behaviours and maternal care patterns observed in the wild at their inshore nursery sites [10,30,31,53]. We consider that our observations of this mother in captivity indicated her positive welfare and that the opportunity to be a mother can give a captive harbour seal female a ‘life worth living’ [60]. Our observations on Victoria and her pups may therefore support an argument in favour of allowing female harbour seals in a zoo setting to give birth, while also having an ethical plan for the long-term welfare of the pups after weaning [21].

It is possible that M’s intense guarding behaviour when suckling P1 (Figure A5) may have indicated anxiety, possibly stemming from her previous two experiences of stillbirths. Her even more extreme guarding behaviour towards the other seals in the group and zoo staff after P2 was born—necessitating her to be confined with P2 to one end of the seal group enclosure—may have been triggered by her previous experiences of stillbirths and of her dying newborn pup being taken from her four years previously. Wild harbour seal mothers bond with their dead newborns and typically carry them around for some days [2,9,61,62], and therefore the necessary removal of a dying neonate in a zoo situation would be expected to leave an emotional scar on the mother. Although a stillbirth or neonatal morbidity would inevitably cause the mother distress, the intense positive effect of a healthy newborn on a captive mother may outweigh that risk.

The harbour seal pups successfully reared by their mothers at Natureland, Skegness, UK, have been released into the wild at approximately four to seven months of age, i.e., about three to six months after the natural weaning age. Questions regarding the positive welfare of these pups post-release must also, therefore, be considered. The exclusive close social contact between a harbour seal mother and pup is believed to be the essence of pups’ primary socialisation and normal brain development [57], and there is no doubt from the video records that both of this captive mother’s pups received this essential maternal contact and care both in quality and quantity. Nevertheless, both pups inevitably lacked the typical orientation experiences, exposure to live prey, and concomitant neurogenesis of wild-born pups [15]. Captive-born pups of spotted seals in China, released at two years of age into the wild, have differed in their spatial distribution and ecological niche from wild seals [21], indicating a potential handicap for young seals released into the wild after this length of time in captivity. We suggest, therefore, that captive-born pups should be released as soon as possible after weaning.

The hope for both captive-bred and rehabilitated pups at Natureland is that after their release to the sea, they will quickly encounter and integrate into the nearby harbour seal colonies in the ‘Wash’ (East England), where approximately 1400 pups are currently born annually [63]. Pup P2 was released ~6 km to the north of the nearest harbour seal haul-out, at the northern edge of the Wash. It is likely that her release companion (rehabilitated male pup ‘Rice’) had a few weeks postnatal orientation to the local coastline before stranding as a post-weaning pup five months previously; he may therefore have retained some memory to aid his orientation away from their release point and towards the nearest harbour seal haul-out. Wild pups at weaning appear to transfer their following response from their mother to other pups [30], and therefore P2’s experience of following her mother closely during fast swimming bursts may have primed her to closely follow Rice out to sea; having a familiar pup as a guide may have helped her to overcome her inevitable lack of sense of place and orientation in a novel marine environment.

## 5. Conclusions

This study found that the range of behaviours of a captive mother harbour seal and her two pups closely resembled that reported for wild mothers and their pups at their nursery sites; we were able to record their underwater behaviours, which would likely be missed in field observations. We discuss the implications of close body contact and underwater body nosing contacts for augmenting a pup’s following response to its mother. The mother in our study had varied options to exert agency in her decisions about where and when she and her pup would rest, haul-out, suckle, and spend time in the water. We conclude that the behaviour of both mother and pups was as natural as possible in the context of captivity, and that the opportunity to give birth and raise her pups with the freedoms observed in this study constituted positive welfare and a ‘life worth living’ [60] for this captive female harbour seal.

## Figures and Tables

**Table 1:** Behaviours described for mother M and pup P1.

Behaviour	Description
*Behaviour of M and P1 in the water*	
Nosing C (contact)	M or P touches the other with nose on any part of the body (including nose-to-nose contact)
Body C	Any part or parts of their bodies in contact (in addition to body nosing)
Twist in C	M and P in close body contact, twisting around each other
P ride	P rides on M’s back
Follow	M or P follows the other as they move forward
Not follow	M or P fails to follow the other as they move forward
Leave	M and P leave when they are together in situ
Approach	M or P approach the other in situ from any distance apart
P swims independently	P swims, apparently without reference to mother
P rests in water	P in situ, apparently asleep, above or below surface
Locomotor play	Leaping (‘porpoising’) out of the water with/without splashing
*Additional behaviours described for M and P1 onshore*	
Signalling to follow	M to P
Suckling initiates	Behaviour of M or P
Suckling terminates	Behaviour of M or P

**Table 2:** Behaviour categories for mother (M) and pup (P2).

Water or Haul-Out	Behaviour Category
Water or haul-out	M or P orients (looks) towards the other
Water	M or P follows the other
Water	P2 dives underneath M
Water	Nose-to-body contact
Water	‘Static’ body contact when stationary
Water	Eyes (M or P2) open, half closed, or closed
Haul-out	Nose-to-body contacts on ‘beach’
Haul-out	Body contact on ‘beach’

**Table 3:** Categories of estimated amount of body contact between M and P2 [31].

Contact Category	Typical Behaviour of M or P2
I	Light touch (e.g., fore-flipper touch)
II	Chin
III	Chin and throat
IV	Chin, throat and chest
V	As above and including mid-venter
VI	Entire pup body in close contact

**Table 4:** No. of snapshots of static body contact between M and P2 in each body contact category.

Contact Category	*N*	I	II	III	IV	V	VI
Water	122	2	5	30	41	25	19
Hauled out	13	0	4	2	6	1	0

**Table 5:** Body regions nosed by M and P1 while idling in the main pool during the early morning.

	Nose-to-Nose	M Noses Head/Neck	M Noses Hind-Flippers	M Noses Shoulder–Rump	P1 Noses Head/Neck	P1 Noses Hind-Flipper	P1 Noses Shoulder–Rump
Surface	23	12	4	6	23	4	5
Underwater	0	0	0	0	2	7	2

**Table 6:** Orientation and behaviour of P2 when following M during rapid swimming.

	*N*	P2 Follows Behind	P2 Follows Hind-Flippers	P2 Follows Side-by-Side	P2 Riding	P2 Nosing Contact
*N*		6	6	9	21	18
						
M at surf	24	2	2	2	17	9
P2 at surf	20	2	0	1	15	7
M uw	20	4	4	6	3	8
P2 uw	23	4	6	7	4	10

*N* = no. occurrences recorded from snapshots; surf = surface; uw = underwater.

**Table 7:** Number of times M approached and contacted P1 while he rested alone onshore.

Day	No. Clips	Total Min	M Approach	M Nosing Contacts	M Body Contact
24 Aug—d3	16	78.5	63	58	4
30 Aug—d9	8	34.65	14	11	0
24 Sept—d3	7	10.79 *	6	0	0

* Visitor presentation with loudspeaker.

**Table 8:** Number of times at least one nosing contact preceded suckling bouts by M and P1.

			No. Nosing Contacts	
	*N*	M Noses P1	Nose-to-Nose	P1 Noses M
M hauls out, P1 follows	16	1	2	5
M & P1 already on beach/pool floor	7	4	4	4
M hauls out to P1	2	1	1	2

*N* = no. suckling bouts in each haul-out context.

## Data Availability

The raw data in the form of spreadsheets are available on request.

## Appendix A

**Table A1:** No. of instances of approaching (app), leaving (leave), and following (foll) by mother (M) and pup P1 in the water of the ‘main pool’ during 30 min CCTV recording in early morning.

P1 Age (d)	M App	M Leave	P App	P Leave	M Foll	P Foll
d1–7 (‘week 1’)	22	25	8	30	37	35
d11–16 (‘week 2’)	27	15	23	51	58	30
Totals	49	40	31	81	95	65

**Table A2:** Suckling bouts by P1: initiation, duration, and terminating. Times in **bold (column 2)** indicate interrupted suckling followed by resumption. Text in ***bold*** (column 5, **Initiate**) indicates where mother initiated suckling resumption following previous interrupted suckling bout. Text in **bold** (column 7, **Terminating behaviour**) indicates where suckling ended when mother was distracted by another female approaching or by a loud noise. M—mother; P—pup; hq—hind-quarters; ho—haul-out; scr—scratches pup with fore-flipper; foll—follow; app—approach; w—water; >—moves; OoS—out of sight; ?—uncertain; shaded rows—pool had been drained.

Day	Time	Length (min)	Location	Initiate	End	Terminating Behaviour
1	06:52	0.35	Birth beach	birth beach, recip. Nosing	M	M shifts hq away from P
1	11:21	3.00	Birth beach	M ho, P foll	M	M > towards water, P foll
1	14:29	19:68	Birth beach	M ho, P foll	M	M > towards water, P foll
1	15:24	2.53	Island	M ho, P foll	P	P detaches, M&P ret to w
1	16:31	0.53	Birth beach	M ho, P foll	P	P detaches, M&P ret to w
1–2	03:50	7.15	Birth beach	M ho, P foll	P	P detaches, M > towards w, P foll
2	17:36	6.13	Pool floor	P seeks nipples, M scr	M	F2 app, M shifts position
2	20:08	4.05	Pool floor	M stretches to P, presents	P	P detaches
2	20:22	3.38	Pool floor	M noses P, presents	M	M shifts position, ending suckling
4	05:51	10.68	Birth beach	M ho to P (P alone on beach)	P?	M OoS, P moving, nosing behaviour OoS
6	08:16	9.08	Birth beach	M ho to P, resting on beach	P?	M OoS, P moves away, nosing behaviour OoS
7	05:00	11.06	Gate beach	M&P already on beach	M	P stopping and restarting, M turns round to terminate
7	09:48	5.43	Birth beach	M ho, P foll	M	M suddenly moves forward
**7**	**17:25**	**1.53**	Birth beach	M ho, P foll	M	**F3 ho, moves behind M&P, M agitated, moves away**
**7**	**17:45**	**11.77**	Birth beach	***M ho, P foll***	M	**F2 ho, app M’s rear, M swings hq away**
7	17:59	1.50	Birth beach	***M lightly scratches P, P > nipples***	P	P detaches, M&P remain on beach
8	**time?**	**5.03**	Birth beach	M ho, P foll	M	**Loud voices, M leans over P, > w P still suckling**
9	**09:57**	**2.53**	Birth beach	M ho, P foll	M	**Loud voices, M raises hq, covers P with ffl, M > w, P still suckling**
9	**17:17**	**4.41**	Pool floor	P seeks nipples	M	**F3 passes by, M lunges at F3**
9	**17:38**	**0.92**	Birth beach via ramp	***M ho via ramp, P foll***	M	**F3 app, M swings round**
9	**time?**	**4.53**	Birth beach via ramp	side-by-side on ramp, P soliciting	M	**F2 app, loudspeaker, M shifts along beach, > w, milk still oozing**
11	05:41	10.75	Birth beach	M ho, P foll	M	M rolls over (P had detached briefly)
12	09:39	5.58	Birth beach	M ho, P foll	M	**F2 app, M turns towards F2, terminates, leans over P**
13	**08:01**	**2.70**	Birth beach	M ho, P foll	M	**F2 app, M shifts towards her**
13	**11:45**	6.30	Island	***M ho, P foll***	M	M > w, terminating, P foll
14	09:25	7.93	Birth beach	M ho, P foll	P	M&P watching F2, P > w as F2 passes
15	**06:04**	**3.74**	Birth beach	M ho, P foll	P	**F3 ho, distracts M, P detaches, >w, M foll**
15	**09:42**	**8.18**	Island	***M ho, P foll***	M	M raises hq, >w, encourages P to foll
16	08:20	6.26	Birth beach	M ho, P foll	P	P detaches when M looks around, P > towards w
